# Recoil-proton track imaging as a new way for neutron spectrometry measurements

**DOI:** 10.1038/s41598-018-31711-z

**Published:** 2018-09-06

**Authors:** Jing Hu, Jinliang Liu, Zhongbing Zhang, Liang Chen, Yuhang Guo, Shiyi He, Mengxuan Xu, Leidang Zhou, Zhiming Yao, Xingqiu Yuan, Qingmin Zhang, Xiaoping Ouyang

**Affiliations:** 1 Department of Engineering Physics and Key Laboratory of Particle and Radiation Imaging, Ministry of Education, Tsinghua University, Beijing, 100084 P. R. China; 2 State Key Laboratory of Intense Pulse Radiation Simulation and Effect, Northwest Institute of Nuclear Technology, Xi’an, 710024 P. R. China; 3 Xi’an Jiaotong University, Xi’an, 710049 P. R. China; 4 Princeton Plasma Physics Laboratory, Princeton University, Princeton, NJ 08543 USA

## Abstract

Recoil-proton track imaging (RPTI) is an attractive technique to optically record the tracks of recoil protons in scintillation gas by using realtime imaging devices. For the first time, its use as an online nuclear track detector for neutron spectrometry measurements (NSM) is explored. Based on the RPTI methodology for NSM, a very basic detector system is designed, consisting of the neutron-to-proton recoil system and proton track imaging system. Satisfactory performance of the RPTI neutron spectrometer has been examined with a series of Monte Carlo simulations. Moreover, using well-defined line-proton sources from a tandem accelerator, the capability of the detector for imaging proton tracks at the single-particle level in real time has been validated in preliminary experiments. From the clear single proton tracks in the images, the proton ranges were easily distinguished, and precise proton energy spectra were unfolded, laying a solid experimental foundation for the future implementation of NSM.

## Introduction

Neutron spectrometry measurements (NSM) have long contributed a lot to the development of nuclear physics^1,2^ and also become an essential component in other important areas, especially fusion plasma diagnostics, radiotherapy and radiation protection^3–7^. Nuclear track techniques^8,9^, typically nuclear emulsion^10^, CR-39^11^ and the fluorescent nuclear track detector (FNTD)^12^, are traditional detectors for NSM, mainly based on the recoil proton method and unfolding the neutron energy after recording numerous tracks of recoil protons. But for reconstructing the nuclear tracks, they involve either laborious chemical processing or post readout using a special microscope after the neutron irradiation. Sometimes, however, the recoil tracks need to be read out in real time so as to determine the neutron spectra promptly for further data analysis or other purposes.

To address this deficiency, recently we have investigated the recoil-proton track imaging (RPTI) method as an online nuclear track technique for NSM. RPTI combines gas scintillation with a realtime imaging device. Using an advanced scientific camera (such as an image-intensified CCD/COMS camera), coupled with a transparent scintillation gas of high photon yield and visible light spectra (such as CF_4_), makes it possible to image the recoil-proton tracks in scintillation gas in real time, analyze the track information online, and further unfold the neutron energy promptly.

Here we systematically describe the methodology of the RPTI technique for NSM and design a very basic RPTI detector system in section 2. Then in section 3, some simulations for the performance of the designed RPTI detector are conducted with a Monte Carlo code. Section 4 reports the preliminary experiments that are performed to test the track imaging system for proton tracks and further proton spectra; in the last section, summaries and future works are discussed.

## Methodology

### Recoil proton method

Collimated incoming neutrons scatter from hydrogen nuclei in a polyethylene (CH_2_) foil, producing recoil protons. In the laboratory coordinate system, the recoil proton energy (*E*_*p*_) is related to the impinging neutron energy (*E*_*n*_) in terms of the scattering angle (*θ*)

1 $${E}_{p}={E}_{n}co{s}^{2}\theta .$$

Thus to measure the neutron energy (*E*_*n*_), we should firstly obtain the energy of the recoil protons (*E*_*p*_) in the scattering direction *θ*.

### Proton track imaging method

Recoil protons deposit their energy in a scintillation gas, such as a noble gas or CF_4_ gas, and generate scintillation photons along their tracks. The spatial distribution of the induced photons indicates the proton track. So if the photons can be recorded by using a realtime imaging device, one can observe the proton track image, from which the proton track range will be extracted.

For charged particles in a given absorber, the specific energy loss (*−dE*/*dx*) is expressed by the Bethe formula^13^

2 $$-{(\frac{dE}{dx})}_{ion}={(\frac{1}{4\pi {\varepsilon }_{0}})}^{2}\frac{4\pi {z}^{2}{e}^{4}e}{{m}_{0}{v}^{2}}NB,$$

with

3 $${\rm{B}}={\rm{Z}}\cdot \,\mathrm{ln}(\frac{2{m}_{0}{v}^{2}}{I}).$$

**Figure 1:**
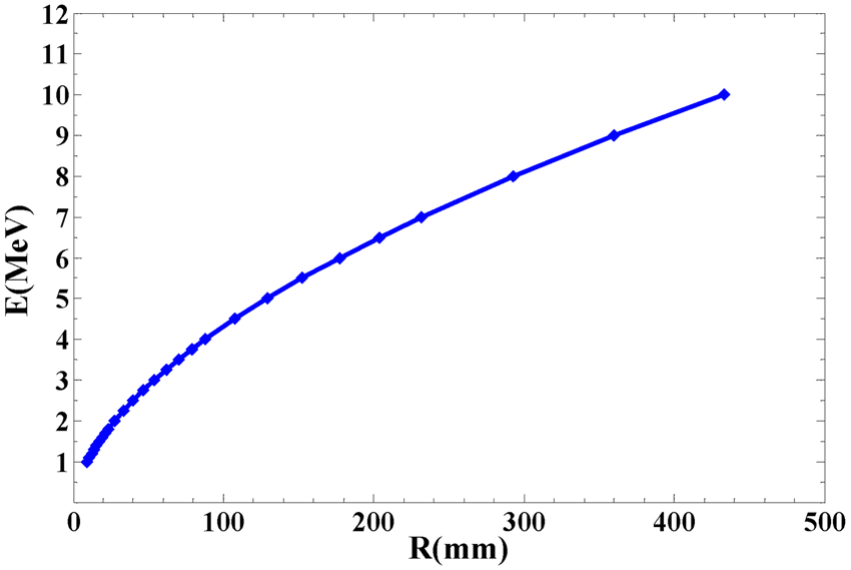
The track Energy- Range relation calculated by SRIM code for 1 MeV to 10 MeV protons in 1 bar CF_4_ gas.

In Eqs (2, 3), *z* and *v* are the atomic number and velocity of the primary particle respectively, *m*_0_ is the rest mass of electron, *Z* and *N* are the atomic number and number density of the absorber atoms respectively, and *e* is the charge of an electron. The parameter *I* represents the average ionization and excitation potential of the absorber, and is experimentally determined for each element. As predicted by Eq. (2), the track range varies with the initial energy. The relationship between proton range (*R*) and initial energy (*E*) of protons in 1 bar CF_4_ gas has been calculated as an example with the SRIM code^14^ and shown in Fig. 1. There is a one-to-one correspondence between the initial proton energy and the proton range.

Thus, with the proton tracks optically recorded as track images, the track ranges can be directly extracted, and then the energy spectrum of the recoil protons can be derived according to the Energy-Range relationship in the given gas (the Energy-Range method). Eventually the neutron spectrum will be unfolded based on Eq. (1).

### Basic detector system

**Figure 2:**
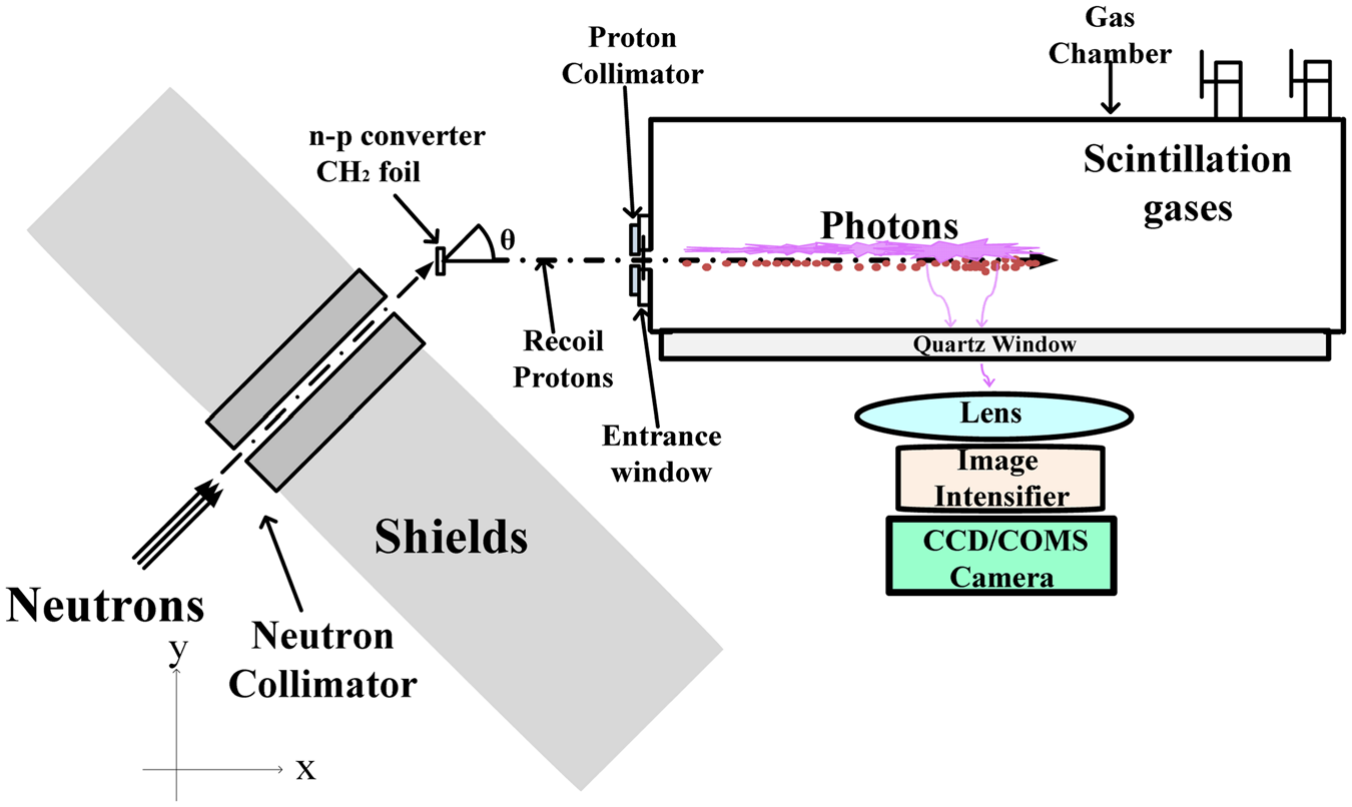
Schematic layout of the designed basic detector systems based on the RPTI method for NSM.

Figure 2 shows a schematic layout of a basic RPTI detector system based on the above methodology. This detector includes two major components. The first component is a neutron-to-proton recoil system, mainly consisting of a neutron collimator and shields, a CH_2_ foil for converting neutrons to recoil protons, and a proton collimator for selecting the incident proton with a well-defined recoil angle *θ*. The second component is a proton track imaging system, including a gas scintillation chamber for producing photons along the proton tracks and a set of imaging devices for recording the proton-track photons as an image.

CF_4_ is our preferred scintillation gas, not just because of its outstanding properties (such as wide spectra wavelength, relatively high yield and fast emission)^15–19^ for imaging purpose, but also for the fact that it is a heavy molecule that contains only low-Z atoms, which is insensitive to gamma radiation and allows a good neutron/gamma discrimination ratio. In engineering design, the gas chamber should have a thin entrance window, such as a 5 μm-thickness Ti window, for sealing the gas while permitting recoil protons through. A quartz window should also be installed to transfer photons to the outside imaging devices. Advanced scientific cameras, such as image-intensified CCD/CMOS, can be chosen as the imaging devices to record the proton-track images in real time, and the acquired imaging data will be transmitted to the online analysis system for unfolding the energy spectrum promptly.

The neutron energy spread (Δ*E*_*n*_) is defined as the full width at half maximum (FWHM) of the neutron energy distribution. For a full RPTI neutron spectrometer based on the design in Fig. 2, Δ*E*_*n*_ can be estimated from

4 $${\rm{\Delta }}{E}_{n}=\sqrt{{\rm{\Delta }}{E}_{C}^{2}+{\rm{\Delta }}{E}_{k}^{2}+{\rm{\Delta }}{E}_{a}^{2}+{\rm{\Delta }}{E}_{w}^{2}+{\rm{\Delta }}{E}_{g}^{2}},$$

where Δ*E*_*C*_ stands for the spread in energy loss in the CH_2_ foil, and depends on the thickness and density of the foil. Δ*E*_*k*_ represents the kinematic energy broadening that results from the scattering angle and its solid angle. Δ*E*_*a*_, Δ*E*_*w*_, and Δ*E*_*g*_ are respectively the energy straggling of the recoil protons in the gap air, thin entrance window and CF_4_ gases, respectively.

Assuming that the collimated protons can be all transmitted into the gas chamber, the detection efficiency of the full neutron spectrometer is calculated by

5 $${\rm{\varepsilon }}({\rm{n}})={N}_{H}\frac{d{\sigma }_{np}}{d{\rm{\Omega }}}{{\rm{\Omega }}}_{s}L,$$

where *N*_*H*_ is the density of the H atom in the CH_2_ foil; *dσ*_*np*_/*dΩ* is the differential cross section for n–p scattering at the recoil angle of (*θ*, *θ* + *dΩ*) *—* into the gas chamber; *Ω*_*s*_ represents the solid angle for accepting the recoil proton, which depends on the air-gap distance and the inner diameter of the proton collimator. *L* is the effective thickness of the converter.

### Simulations of the performance

In order to evaluate the performance of the RPTI method for NSM, a series of Monte Carlo simulations^20^ have been performed using the configuration in Fig. 2. Taking DT neutron measurements (very common in many applications) as an example, the collimated neutron source is defined as a neutron beam at 14 MeV. The direction of the neutron beam is set as 45 degrees from the x-axis, which is chosen here just for simplicity in the analysis. In practical applications, this angle should be carefully selected to reduce the direct neutron radiation. The incident neutrons directly impinge on a CH_2_ foil, and generate recoil protons. The effective area (hit by the collimated neutron beam) of the CH_2_ foil is π × 0.5^2^ cm^2^. A Fe disk with thickness of 2 mm and inner radium of 3 mm is used as a proton collimator, positioned at 1 mm away from the entrance window (5 μm-thickness Ti window here). The protons along the x-axis direction are selected to enter the gas chamber, which is filled with 1 bar CF_4_ gases. The distance between the CH_2_ foil and proton collimator is set to be 42 mm.

**Figure 3:**
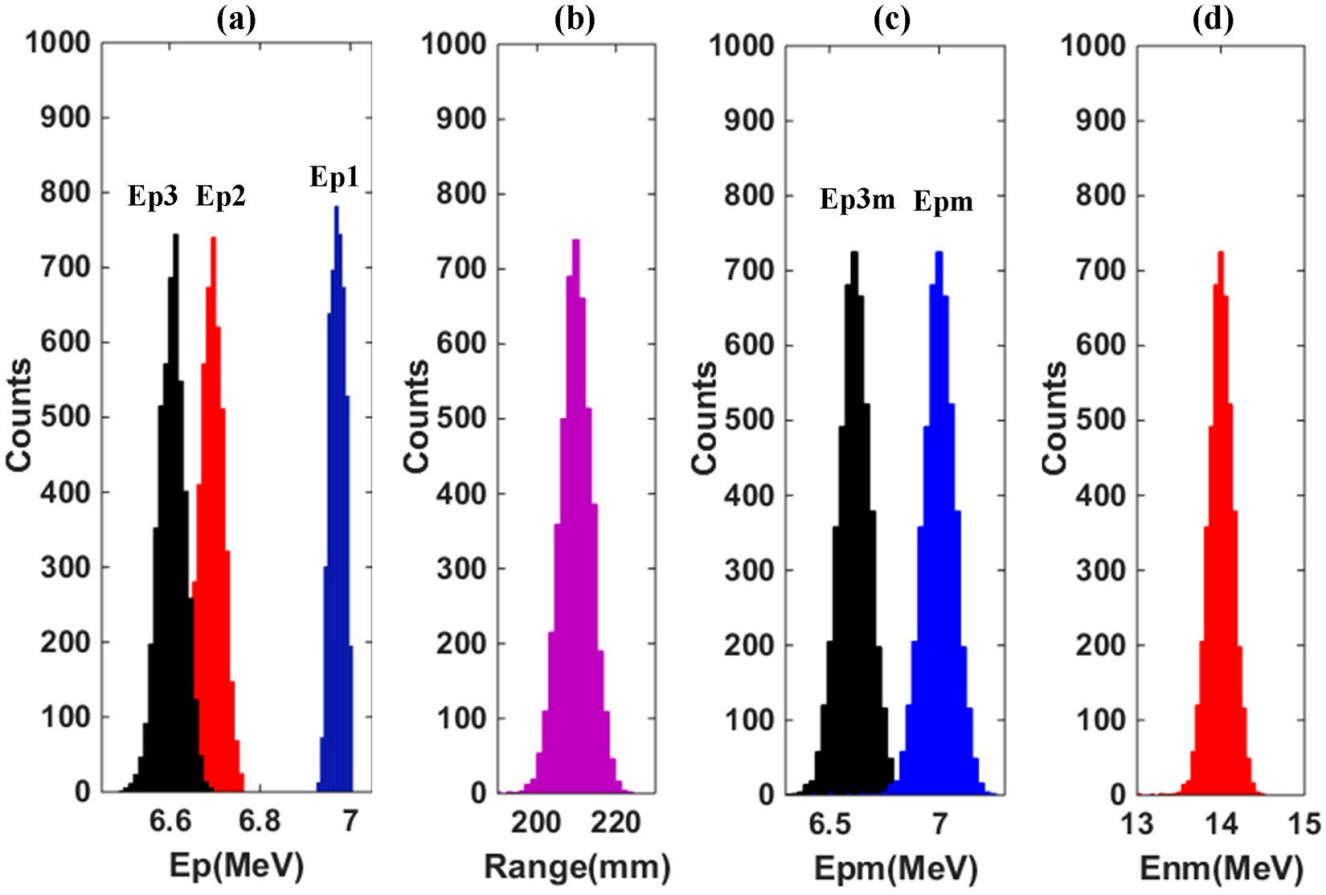
Calculated results with Monte Carlo simulations. (**a**) Recoil proton energy spectra after passing the CH_2_ foil (Ep1), the gap air (Ep2), and the Ti window (Ep3); (**b**) proton range distribution in the CF_4_ gas; (**c**) the unfolded initial proton spectrum into CF_4_ gas (Ep3m) and recoil proton spectrum (Epm) from n-p scattering; (**d**) the neutron spectrum (Enm) calculated from the Epm by means of Eq. (1), using a CH_2_ foil with density of 0.94 g/cm^3^ and thickness of 10 μm.

The simulation records the residual proton energy after passing the CH_2_ foil (represented by Ep1), the air gap (represented by Ep2), and the Ti window (represented by Ep3), respectively. The energy spreads resulting from the five factors in Eq. (4) are all considered in the simulation and the results are shown in Fig. 3, where a CH_2_ foil with density of 0.94 g/cm^3^ and thickness of 10 μm is used. Figure 3(a) presents the detailed spectrum broadening of protons in each passing medium (CH_2_, air, and Ti) and it is seen that all proton spectra are approximately Gaussian in form. Figure 3(b) shows the resulting proton range distribution, from which the initial energy distribution of the protons before entering the CF_4_ gas chamber [Ep3 m in Fig. 3(c)] is derived based on the Energy-Range relation in 1 bar CF_4_ gas. Combined with the Energy-Range relations in Ti and air (calculated by SRIM code), and also corrected for the average energy loss in the CH_2_ foil, the energy spectrum of recoil protons [Epm in Fig. 3(c)] is also acquired. Finally, the neutron spectrum [Enm in Fig. 3(d)] is unfolded from the recoil proton energy spectrum by means of Eq. (1).

The average value and FWHM corresponding to each spectrum in Fig. 3 are listed in Table 1. It is revealed that the unfolded neutron spectrum has an average energy (peak energy) of 14 MeV with a FWHM of 326 keV. The simulated neutron energy resolution (FWHM/E) of the whole system is 2.33% at 14 MeV. If the measurement system setup is fixed, the response function from the recoil proton range distribution to the neutron spectrum is a known factor based on the above unfolding processes [from Fig. 3(b–d)].

**Table 1:** The characteristics of each spectrum in Fig. 3.

	Ep1 (MeV)	Ep2 (MeV)	Ep3 (MeV)	Range (mm)	Epm (MeV)	Enm (MeV)
Average	6.969	6.693	6.609	209.9	7	14
FWHM	0.0365	0.0615	0.0676	8.311	0.163	0.326

**Figure 4:**
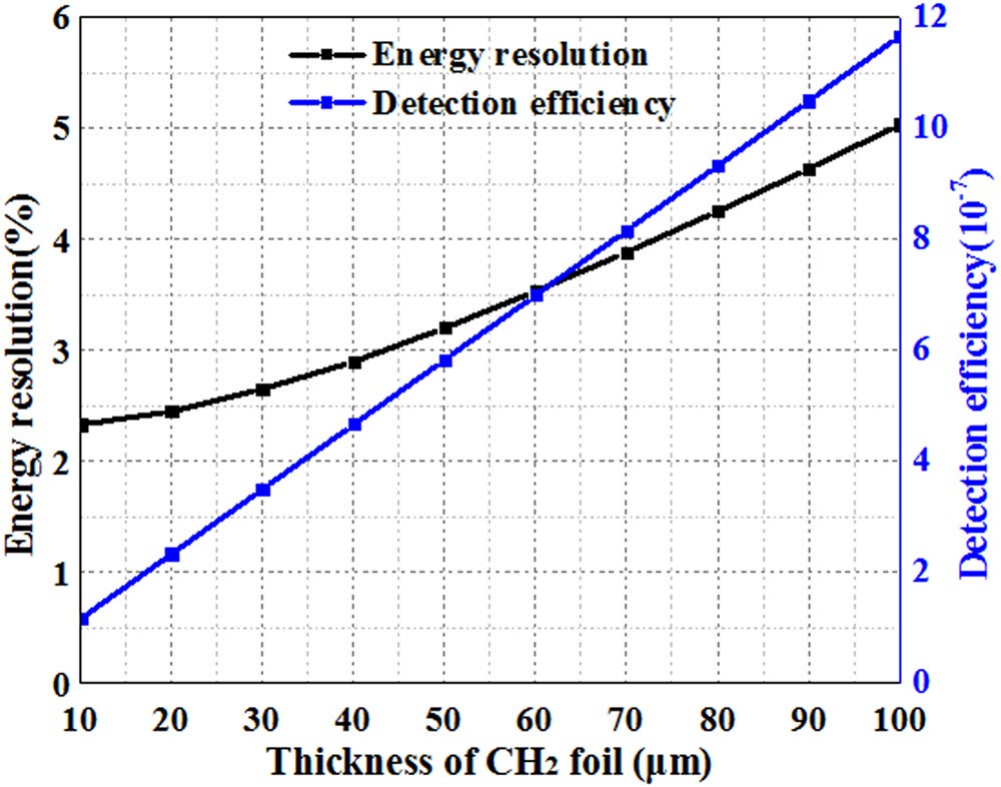
The simulated energy resolution (black line) and efficiency detection (blue line) as the function of the thickness of the CH_2_ foil.

The neutron energy resolution and detection efficiency were also computed as a function of the thickness of the CH_2_ foil. The detection efficiency is defined by the ratio of the total number of protons into the gas chamber to the total number of incident collimated neutrons. An energy resolution (FWHM/E) of 2.33% for 14 MeV neutrons can be achieved with a detection efficiency in the order of 10^−7^. The calculated results in Fig. 4 show that as the thickness of the CH_2_ foil increases, the neutron detection efficiency becomes higher, but the energy resolution gets worse. In practical applications, the thickness of CH_2_ can be flexibly changed to adjust the energy resolution and neutron efficiency. Also, the solid angle of recoil protons has a similar effect on the tradeoff between the energy resolution and the detection efficiency, and can be adjusted by altering the diameter of the proton collimator, the distance between the CH_2_ and proton collimator, or the neutron impacting area of CH_2_ foil. In the high-yield measurements, it is better to operate the system in a high-resolution mode by using either a thinner CH_2_ or a smaller solid angle of recoil protons. However, for low-yield cases, as the detection efficiency is critical to normal operation, it is necessary to increase the CH_2_ thickness or enlarge the proton solid angle appropriately at the cost of a degraded energy resolution.

### Preliminary experiment setup

The preceding discussion of RPTI for NSM assumed that it is possible to measure proton ranges accurately. Before testing its use with neutrons, a first priority is to examine the performance of the proton track imaging system for proton tracks using protons of well-defined energy. Preliminary experiments have been carried out at the EN-6 tandem accelerator at the Institute of Heavy Ion Physics, Peking University.

**Figure 5:**
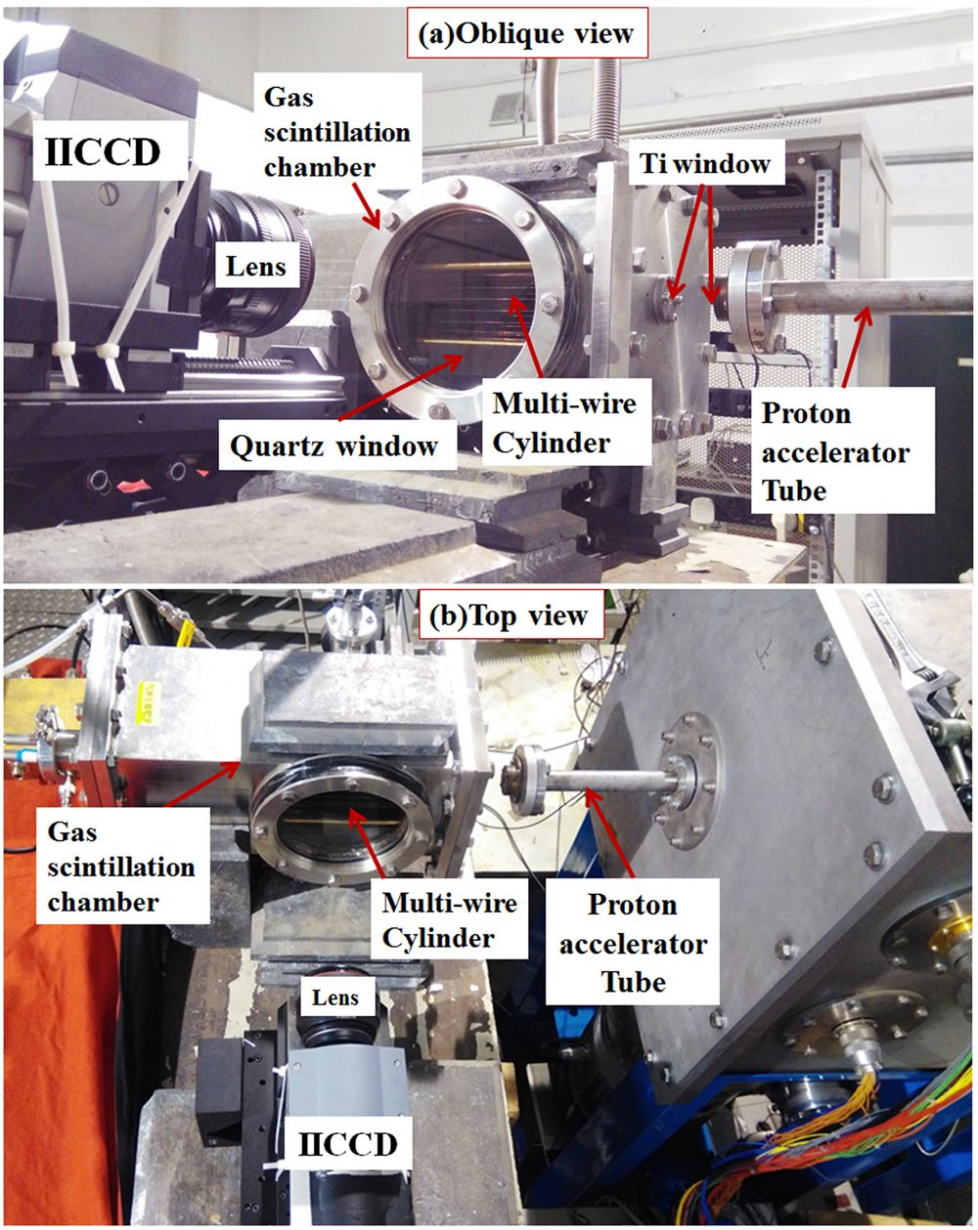
Experimental setup for preliminary test of the RPTI method with protons from accelerator at (**a**) Oblique View and (**b**) Top view.

As shown in Fig. 5, the experimental setup consists of a proton accelerator and the proton track imaging system. The tandem accelerator can deliver protons with energy ranging from 2 MeV to 10 MeV by varying the terminal voltage. In these experiments, protons of 5.91 MeV and 6.30 MeV were delivered from the accelerator. As shown in Fig. 5, before entering the gas scintillation chamber, the delivered protons passed through a Ti window (5 μm in thickness) installed on the exit of the accelerator tube, an air gap of 45 mm, and another Ti window (also 5 μm thickness) at the entrance of the gas chamber.

The gas scintillation chamber was filled with 1 bar CF_4_ gas. The total scintillation yield of CF_4_ is about 2000 photons/MeV (measured with alpha-particles) at 1 bar^17^. For an individual proton, the number of induced primary photons in CF_4_ are too small to be detected by any scientific cameras available to us. To address this issue, a strong electric field can be utilized to generate photon avalanches^21,22^, which produces a large population of photons that are able to be optically recorded. It should be noted that this kind of electric field can be easily achieved by using a proper electrode geometry inside the gas chamber, such as the gaseous detectors^23^ like Optical Avalanche Chamber (OPAC)^24^, Micro-Strip Gas Chambers (MSGC)^25^, Gas Electron Multipliers (GEM)^26^ or other simple structure. For this initial proof-of-principle experiment, we fabricated a simple multi-wire cylinder structure, which is consisted of 18 cathode copper wires with 100 μm in diameter, and an anode gold-plated tungsten wire with 25 μm in diameter. The 18 cathode wires are circular uniformly distributed around the anode wire with 30 mm in distance between the anode wire and each around cathode. It is similar to a cylindrical proportional counter^27^, but with dozens of cathode wires instead of the cathode tube, thus the cathode-wire gaps are able to transport the light outside. In order to get the proton-track ranges, the direction of incident protons was parallel to the anode wire. This ensured that the avalanche-induced photons became the parallel projection of the proton track in this electric field, and retained all the required longitudinal information of the track for extracting proton ranges. The scientific camera we used here is an image-intensified CCD (IICCD), with 1024 × 1024 active pixels (each pixel size 13 × 13 μm^2^).

## Experimental Results and Discussions

### Recorded proton track images

**Figure 6:**
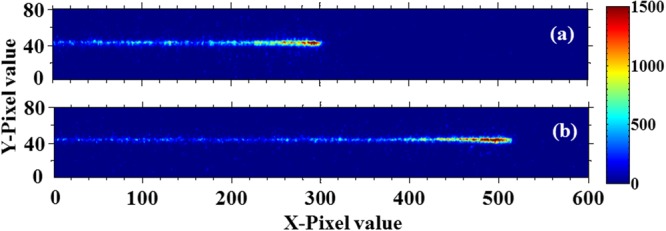
The typical single proton track images for (**a**) 5.91 MeV and (**b**) 6.30 MeV delivered proton in 1 bar CF_4_ gas with a strong electric field. The protons incident direction is from left to right and the color scales are the relative intensity (gray value) in the images.

With the cathode wires grounded while the anode wire was supplied with +3000 V (light gain about 300) in the gas chamber, a strong electric field (of the order of 10^7^ V/m)) was produced in the vicinity of the anode, where avalanche-induced photons from individual proton tracks were generated and then successfully recorded by the IICCD. Typical single proton track images for 5.91 MeV and 6.30 MeV protons in 1 bar CF_4_ gas are shown in Fig. 6(a,b) respectively, where the color scale represents the relative intensity. It is revealed that the proton tracks on the images are very distinguishable, especially near the termination of the track, which is the region of interest for extracting ranges. In this experimental system, the track range (*R*/mm) is linear to the terminal position (expressed by pixel value *N*_*p*_) of recorded track on the images as

6 $$R=130(mm)+{N}_{p}\times 76(mm)/1024$$

where 76 mm is the size of the imaging view, 130 mm is the distance from the left side of the imaging field to the Ti window of the gas chamber, and 1024 is the number of CCD pixels. *N*_*p*_ can be retrieved backwards from the end of the image, exactly at the first X-pixel position where the average intensity (gray value) is nearest to 10% of the maximum intensity. The *N*_*p*_ values extracted from Fig. 6(a,b) are 300 and 512, respectively, and thus based on the above linear conversion, the proton ranges are 152 mm and 168 mm, respectively.

### Unfolded proton energy spectrum

**Figure 7:**
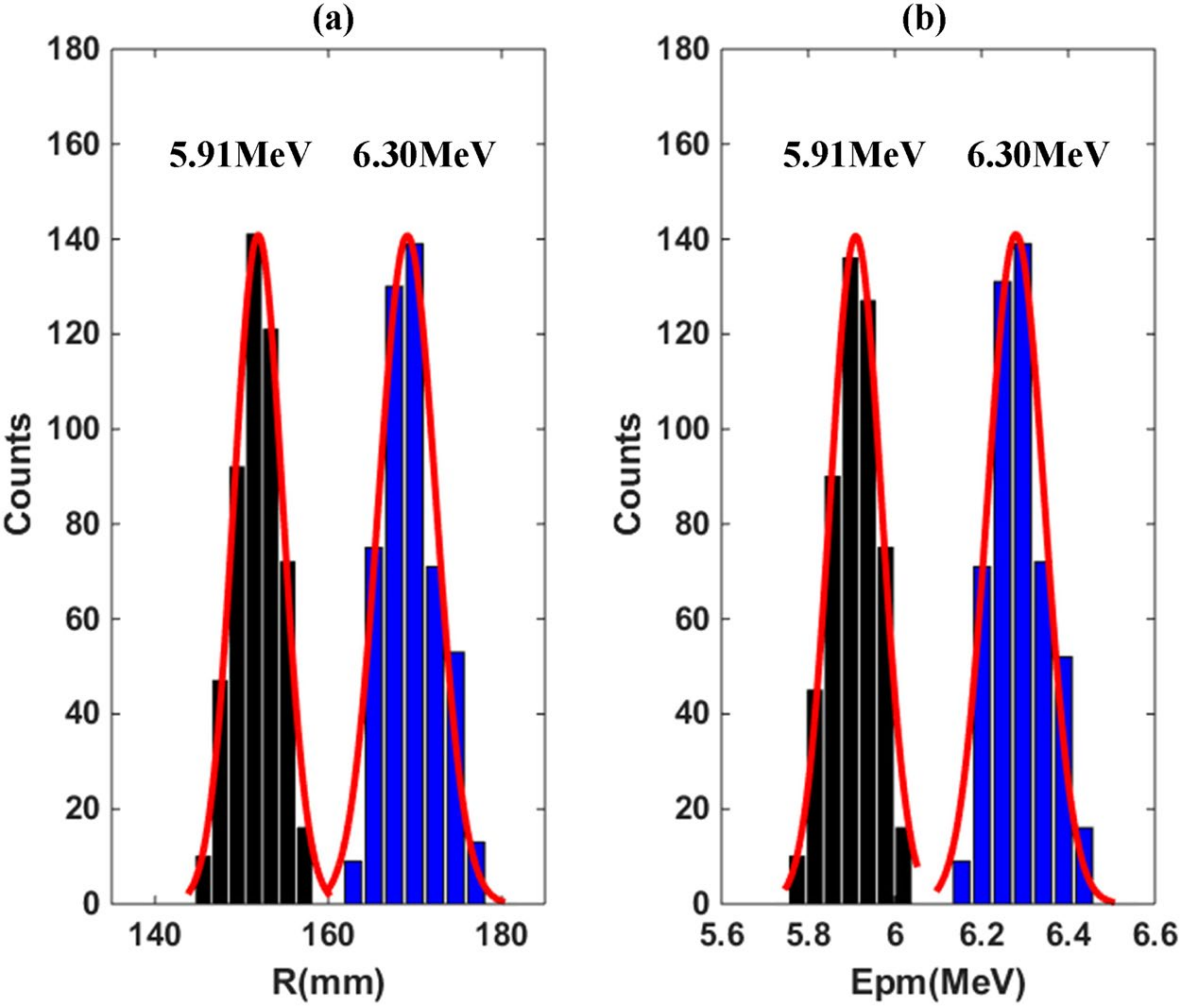
(**a**) The range distributions of single proton in 1 bar CF_4_ gas for 5.91 MeV and 6.30 MeV delivered protons; (**b**) The reconstructed proton spectra from the ranges in the left figure.

In the experiments, approximate 500 single proton track images at the nominal energies of 5.91 and 6.30 MeV from the accelerator were respectively acquired to reconstruct the calibration spectra. The range distributions of the single proton tracks are shown in Fig. 7(a), in which fitted Gaussian distributions (red solid curves) are also plotted. The characteristics for the two different incident energies are listed in Table 2, including the mean ranges (*R*_*m*_) and the FWHM of the range. The measured range deviation is determined by range straggling and the transversal diffusion of electrons during the drift motion. Mono-energetic protons experience track range straggling is theoretically in single-Gaussian distribution. Primary electrons induced by protons drift towards the avalanche regions, and might spread in the direction perpendicular to the drift. The diffusion can be reduced by decreasing the length of the drift path.

**Table 2:** The distributional characteristics of the proton ranges and spectra corresponding to Fig. 7(a,b).

*E*_*p*_ MeV	*R*_*m*_ (FWHM) mm	*E*_*pm*_ (FWHM) MeV
5.91	151.9 (6.52)	5.90 (0.137)
6.30	169.1 (7.58)	6.28 (0.150)

After each proton track was imaged, the proton energy can be promptly calculated from the extracted range according to the track Energy-Range relations in the passing materials (1 bar CF_4_ gas, a 5 μm-thickness Ti window, an air gap of 45 mm, and another 5 μm-thickness Ti window). The unfolding process is very similar to the process described in the simulation and can be automatically performed using a simple program. All of the calculated proton energies form the measured proton spectra, shown with histograms in Fig. 7(b). The unfolded proton spectra agree well with the single Gaussian distributions (red solid curves) as expected. The energy spectra parameters extracted from Fig. 7(b) are also listed in Table 2. The measured peak proton energies (*E*_*pm*_) are 5.90 and 6.28 MeV, and the FWHM of the spectra are 0.137 and 0.150 MeV, resulting in the energy resolutions (FWHM/*E*_*pm*_) of 2.32% and 2.39%, respectively for the nominal energies of 5.91 and 6.30 MeV. The peak energies are slightly smaller than the corresponding nominal energies, which probably results from the errors of measurement in the Ti thicknesses, air gap and gas pressures. The energy broadening in the proton spectra is mainly caused by the range straggling in the CF_4_ gas, and the energy spreads in the gap air and the two Ti windows also contributed. In general, precise proton energy spectra are successfully unfolded from the recorded track images, and the capability of the proton track imaging technique is well demonstrated.

## Conclusions and Future Works

In this paper, an online nuclear track technique for NSM based on RPTI methodology has been developed, with the advantageous functions of imaging single proton tracks in real time and further unfolding the neutron spectra online. By using Monte Carlo simulations, the designed RPTI detector was evaluated with good performance for NSM and the energy resolution (FWHM/E) of 2.33% at 14 MeV can be achieved with a detection efficiency in the order of 10^−7^. Particularly, the crucial track imaging capability was tested experimentally using protons of well-defined energy from a tandem accelerator. A number of clear tracks at single-proton level were successfully imaged, indicating high sensitivity of the system. Most importantly, the proton ranges were easily distinguished from the track images and further used to unfold the proton energy spectra, resulting in energy resolutions (FWHM/*E*) of 2.32% for 5.91 MeV protons and 2.39% for 6.30 MeV protons. Thus, the practical use of the proton track imaging system has been demonstrated, which paves the way for the next study on NSM.

On this basis, neutron measurements will be performed once a suitable neutron source is available to our team. The general properties, along with the aforementioned features of the RPTI technique for NSM will be further investigated experimentally.
